# Reply to Daitch et al. Comment on “Huang et al. Colistin Monotherapy versus Colistin plus Meropenem Combination Therapy for the Treatment of Multidrug-Resistant *Acinetobacter baumannii* Infection: A Meta-Analysis. *J. Clin. Med.* 2022, *11*, 3239”

**DOI:** 10.3390/jcm11247508

**Published:** 2022-12-19

**Authors:** Chienhsiu Huang, Ihung Chen, Tiju Tang

**Affiliations:** Department of Internal Medicine, Dalin Tzu Chi Hospital, Buddhist Tzu Chi Medical Foundation, NO. 2, Min-Sheng Road, Dalin Town, Chiayi 62247, Taiwan

This is an Author Reply to the Letter to the Editor entitled “Colistin Monotherapy versus Colistin plus Meropenem Combination Therapy for the Treatment of Multidrug-Resistant *Acinetobacter baumannii* Infection: A Meta-Analysis” by Daitch V. et al. [1].

First, the meta-analysis should exclude unpublished data. Therefore, we excluded the clinical trial “Trial for the Treatment of Extensively Drug-Resistant gram-negative Bacilli (OVERCOME)” by Kaye K. et al. [2].

Second, the study of Paul M. et al. entitled “Colistin alone versus colistin plus meropenem for treatment of severe infections caused by carbapenem-resistant gram-negative bacteria: an open-label, randomized controlled trial” included 151 colistin monotherapy cases versus 161 colistin plus meropenem therapy cases [3]. The study of Dickstein Y. et al. entitled “AIDA Study Group. Treatment Outcomes of Colistin- and Carbapenem-resistant *Acinetobacter baumannii* Infections: An Exploratory Subgroup Analysis of a Randomized Clinical Trial” included 135 colistin monotherapy cases versus 131 colistin plus meropenem therapy cases [4]. The study of Nutman A. et al. entitled “Colistin plus meropenem for carbapenem-resistant gram-negative infections: in vitro synergism is not associated with better clinical outcomes” included 136 colistin monotherapy versus 131 colistin plus meropenem therapy cases [5]. The total numbers of populations and objectives of the studies were different among the three studies. We failed to note that the study by Dickstein Y. et al. [4] and the study by Nutman A. et al. [5] are subgroup analyses of the study by Paul M. et al. [3] We would like to thank Dr. Daitch V. [1] for informing us of this error. Therefore, our meta-analysis when corrected includes one randomized controlled trial and seven retrospective studies. We will exclude these two noted studies, and thus need to perform a correct analysis of efficacy outcomes, including clinical improvement, 14-day mortality, and hospital mortality.

Regarding the clinical improvement, eight studies involving 1217 patients (524 with colistin monotherapy, 693 with colistin plus meropenem combination therapy) reported clinical improvement. There was no statistically significant difference in clinical improvement between patients treated with colistin monotherapy and colistin plus meropenem combination therapy (OR = 0.85, 95% CI = 0.66–1.10, *p* = 0.22, I^2^ =19%) (Figure 3) [6].

Regarding the 14-day mortality, four studies involving 682 patients (305 with colistin monotherapy, 377 with colistin plus meropenem combination therapy) reported 14-day mortality. There was no statistically significant difference in 14-day mortality between patients treated with colistin monotherapy and colistin plus meropenem combination therapy (OR = 1.12, 95% CI = 0.81–1.55, *p* = 0.50, I^2^ = 48%) (Figure 5) [6].

Regarding the hospital mortality or 28-day mortality, six studies involving 1003 patients (461 with colistin monotherapy, 542 with colistin plus meropenem combination therapy) reported hospital mortality or 28-day mortality. There was no statistically significant difference in hospital mortality or 28-day mortality between patients treated with colistin monotherapy and colistin plus meropenem combination therapy (OR = 1.04, 95% CI = 0.67–1.62, *p* = 0.86, I^2^ = 62%) (Figure 6) [6].

Conclusions: The current meta-analysis found that colistin monotherapy was associated with similar rates of clinical improvement, 14-day mortality, and hospital mortality to colistin plus meropenem combination therapy. The analysis of efficacy outcomes is the same as in our previous meta-analysis [6].

## Data Availability

The datasets generated during and/or analyzed during the current study are not publicly available but are available from the corresponding author on reasonable request.
